# Tailored Sailing Experience to Reduce Psychological Distress and Improve the Quality of Life of Breast Cancer Survivors: A Survey-Based Pilot Study

**DOI:** 10.3390/ijerph17124406

**Published:** 2020-06-19

**Authors:** Daniela Mirandola, Giovanna Franchi, Alice Maruelli, Manuela Vinci, Maria Grazia Muraca, Guido Miccinesi, Mirko Manetti, Mirca Marini

**Affiliations:** 1 Department of Experimental and Clinical Medicine, Section of Anatomy and Histology, University of Florence, 50134 Florence, Italy; daniela.mirandola@unifi.it (D.M.); mirko.manetti@unifi.it (M.M.); 2 Oncological Rehabilitation Center (Ce.Ri.On.), 50139 Florence, Italy; g.franchi7@gmail.com (G.F.); alicepamaruelli@gmail.com (A.M.); m.muraca@ispro.toscana.it (M.G.M.); 3 The Italian League against Tumors (LILT), 50126 Florence, Italy; manuela.vinci@stud.unifi.it; 4 Oncological Network, Prevention and Research Institute (ISPRO), 50139 Florence, Italy; g.miccinesi@ispro.toscana.it

**Keywords:** sailing, breast cancer, cancer survivors, quality of life, psychological distress, psychological well-being

## Abstract

*Background*: Growing evidence indicates that physical/sporting activities may improve the health outcomes and quality of life (QoL) of breast cancer (BC) survivors. Since recent reports have suggested that sailing can improve the psychophysical well-being and QoL of people with disabilities, this pilot study evaluated the effectiveness of a tailored sailing experience on the QoL and psychological distress (PD) of BC survivors. *Methods*: A group of 19 breast cancer survivors, who were attending the Cancer Rehabilitation Center in Florence, were invited to participate in a sailing school and completed a survey based on a structured online questionnaire assessing QoL and PD both on departure (baseline) and one week after returning (follow-up). The survey comprised a first part (i.e., sociodemographic characteristics and the practice of physical/sporting activities at baseline; sailing experience satisfaction at follow-up) and a second part (i.e., Short Form-12 (SF-12), State/Trait-Anxiety Inventory form Y (STAI-Y), distress thermometer questionnaires). A paired Student’s *t*-test was used to compare the baseline versus follow-up QoL and PD scores. *Results*: A statistically significant improvement in SF-12 mental component scores and a reduction in both STAI-Y state/trait components and distress thermometer scores were found after the sailing experience. *Conclusions*: We conclude that sailing practice could be a feasible intervention to increase the psychophysical well-being of BC survivors.

## 1. Introduction

Growing evidence indicates that physical or sporting activities may improve the health outcomes and quality of life (QoL) of breast cancer (BC) survivors [1,2,3]. In fact, even though survival rates are continually increasing, BC treatments often cause long-term physical sequelae and psychological distress (PD) [4]. The main complications of BC treatments are represented by a reduction in the strength and mobility of the upper limbs and the presence of lymphedema, pain and postural instability, with a consequent alteration of body image and production or exacerbation of emotional disturbances (i.e., mood disorder, anxiety, depression, and anger) which negatively influence QoL [1,2,4]. Moreover, the fear of cancer recurrence and the distress associated with follow-up examinations can also aggravate mood issues [2]. Therefore, BC survivors should be encouraged to initiate and maintain a physically active lifestyle [1,2,3]. However, a high number of BC survivors do not adhere to international clinical practice guidelines in terms of healthy lifestyles, and therefore it is of primary importance to search strategies to promote lifestyle behavior changes [5]. It is well known that physical and mental health, social functioning and emotional well-being and, consequently, a better QoL may be derived from the practice of physical activity and/or sports [6]. In particular, there is evidence that participation in team sports rather than individual activities may be associated with better health outcomes, due to the social nature of the participation [7,8]. At present, research exploring the relationship between the team-based physical/sporting activities and the QoL of BC survivors is limited to dragon boat racing [9,10]. In addition, Tai Chi and Qigong, which are two increasingly popular mind–body interventions typically delivered in groups, have been shown to provide multiple physical and psychosocial benefits for cancer survivors [11]. Similarly, dance practiced in groups can represent an effective and valid tool to ameliorate the psychophysical wellness of BC survivors [12]. Recently, it has also been reported that higher physical activity levels are associated with a better QoL among BC survivors. Therefore, oncologists should closely collaborate with physical exercise specialists in order to elaborate appropriate and effective strategies to increase the physical activity level of cancer survivors [13].

Interestingly, it has recently been proposed that the practice of sailing may be useful to improve the psychophysical well-being and QoL of people with disabilities [14,15,16,17]. Sailing is a complex activity that involves the perception/integration of both exteroceptive and proprioceptive stimuli owing to the fact that it is practiced in a stimulating environment, such as the sea or a lake [16]. Indeed, sailing is considered a practical and physically safe sport for disabled people that may exert positive effects on self-esteem and general health, possibly becoming an integrated part of rehabilitation [14,15,16,17]. In particular, people with severe mental disorders and psychosocial disabilities who participated in a structured sailing program showed a statistically significant improvement in their QoL and clinical status [14,15]. Furthermore, sailing has been reported to improve the QoL of disabled children and adolescents with neurological impairments in motor coordination and balance [16]. Finally, another recent study demonstrated a positive psychological impact of sailing practice among Veterans with substance use disorders [17]. However, the possible positive effects of this sport on the QoL and PD of cancer survivors have yet to be demonstrated.

The present survey, based on a structured online questionnaire, was therefore undertaken to investigate for the first time whether a tailored sailing experience may significantly improve the QoL and PD of BC survivors.

## 2. Materials and Methods

### 2.1. Participants

A group of 19 BC survivors (all women; mean ± SD age, 53.6 ± 5.9 years, range 43–68 years), attending the Oncological Rehabilitation Center (Ce.Ri.On.) in Florence, were invited to participate in a sailing school and completed a survey based on a structured online questionnaire assessing QoL and PD both on departure (baseline) and one week after returning (follow-up). The median time from BC surgery to sailing experience was 4 years (range, 1 to 23 years). The eligibility criteria were a completed BC treatment (i.e., surgery with an eventual axillary node dissection, chemotherapy and/or radiotherapy, and eventual breast plastic surgery), psychophysical disease-stabilized outcomes and a medical certificate to participate in non-competitive sports. The exclusion criteria were any medical conditions which could interfere with their participation in the sailing activity, non-swimmers, known or suspected BC complications (e.g., distant metastases, local cancer recurrence, upper limb lymphedema, presence of venous access port system), cardiovascular diseases and an age of over 70 years. All consecutive patients, corresponding to the inclusion criteria and without any of the exclusion criteria, attending the center for psychological rehabilitation in the period between January and April 2018, were invited. Since in such a reference period no male patient was attending the center, all the invited BC survivors were women and accepted to take part in the sailing experience. The enrollment was stopped when the planned number of BC survivors, sufficient to compose three small “teams” for a tailored sailing course, was reached. In particular, the 19 participants were divided into three teams consisting of 6 women (team no. 1), 6 women (team no. 2) and 7 women (team no. 3), respectively. The online survey was approved by the Ce.Ri.On. review board. An informed consent form describing the aims and potential risks of the study was collected from all the participants who agreed to have their data, aggregated and anonymized, published in research articles.

### 2.2. Intervention

The tailored sailing experience, aimed at improving the overall QoL of BC survivors, was based on learning to sail in a crew by attending a one week course. The course took place in May 2018 for team no. 1, June 2018 for team no. 2 and September 2018 for team no. 3 in the sailing school at the Sailing Center of Levante (Palau, Sardinia, Italy), where in addition to the cabin cruisers, the participants shared common spaces. Of note, BC survivors were followed by an integrated multidisciplinary group consisting not only of skippers and sailing teachers, but also of two psychologists and a physical exercise specialist from the Ce.Ri.On. staff. The program included both morning theory lessons (e.g., essential right-of-way sailing rules, position of crew, direction of wind, and sailing knots) and a navigation practice phase in the open sea aboard the cabin cruisers (i.e., Nytec 20 “Stark”, Nuova Nytec S.r.l., Milan, Italy) carried out from 2 to 6 p.m. Furthermore, at the end of the daily activity, psychological group sessions were conducted to encourage BC survivors to manifest their impressions and feelings about the group experience, to improve their social and communication skills, self-esteem and self-confidence, promoting both a sense of belonging and a supportive and stimulating environment. The physical exercise specialist supervised the sailing phase and was supportive for every individual need related to technical gestures and movements. Stretching was proposed at the end of navigation to improve participants’ own body knowledge and awareness. The costs of the sailing experience were largely covered by the OLA—OltreLeAli Association (Florence, Italy) in collaboration with the Italian League Against Tumors (LILT, Florence, Italy) and, therefore, the participants had to pay a minimum amount.

### 2.3. Questionnaire

The online survey questionnaire was created in the Italian language using the Google Forms platform [7] and was sent by e-mail to the participants (all Italian native speakers) in order to provide direct access to the online questionnaire. All participants were fluent in Italian. The responses were completely anonymous and confidential. The baseline survey consisted of a first part concerning sociodemographic characteristics including age, educational status, job position and current or previous practice of physical/sporting activities including those proposed by the Ce.Ri.On. center. Any previous experience of navigation and motivation for participation in sailing school were also investigated. The second part included the Short Form-12 (SF-12) questionnaire to evaluate QoL, as well as the State/Trait-Anxiety Inventory form Y (STAI-Y) and distress thermometer questionnaires to assess PD. In particular, the validated Italian version of STAI-Y consists of 20 items to assess state anxiety (i.e., how respondents feel at this moment) and 20 items to evaluate trait anxiety (i.e., how respondents generally feel). Each STAI-Y item is given a weighted score of 1 to 4, and both state and trait components can vary from 20 to 80. A predictive threshold value of anxious symptomatology is set at 40 [18]. The normative population tested with this questionnaire included 2363 Italian working people, sampled regardless of their health-related status [19,20]. The distress thermometer is a visual graphic scale consisting of 11 points with a range from 0 (no distress) to 10 (extreme distress). Each subject indicated their personal level of distress over the course of the week prior to completing the questionnaire [21,22,23]. In brief, the distress thermometer is a simple instrument, used according to the National Comprehensive Cancer Network guidelines on distress management, which has optimal sensitivity and specificity in detecting psychological disorders. The distress thermometer was validated in a large sample of Italian cancer patients, and its utility for oncological patients has been reported frequently in the literature [22,24]. Details on the SF-12 questionnaire have been described elsewhere [1,2,12,25,26]. Briefly, the SF-12 questionnaire has previously been validated in an Italian population showing a good validity [19,26,27]. It consists of 12 items which generate two components: a physical component score and a mental component score. Higher scores on these subscales are indicative of a better QoL. The normative population tested with the SF-12 questionnaire included 61,434 Italian subjects randomly sampled from the electoral lists, regardless of their health status [19,26,27]. Therefore, the values of this group represent the average of the health-related QoL for the general Italian population. In the follow-up survey, the second part remained unchanged while the first investigated the sailing experience satisfaction. In particular, to assess the emotional ratings of subjects on the sailing experience, a three-point scale with standard smiley faces as visual cues was used (i.e., a green happy face corresponded to a positive experience, a yellow neither happy nor unhappy face corresponded to a neutral experience, and a red sad face corresponded to a negative experience). Smiley faces were used in combination with the aforementioned explanatory text labels. Indeed, web surveys allow researchers to use graphic or symbolic elements alongside the text of response, and among them smiley faces can be easily used to communicate positive and negative domains [28,29]. Moreover, smiley face scales are not only easy to use, but also represent a way to make surveys more enjoyable regardless of age [28,29]. Study participants were also requested to indicate if they would repeat the sailing experience (yes/no response options). In addition, the subjects were asked to report if the experience had been a strenuous physical activity (yes/no response options). Finally, participants were asked to indicate whether the sailing experience corresponded to their expectations (yes/no response options).

### 2.4. Statistical Analysis

A paired Student’s *t*-test was used to compare the baseline versus follow-up scores of QoL and PD. A *p*-value < 0.05 was considered statistically significant. Effect size (Cohen’s d) was estimated for all *t*-test analyses. SPSS version 25.0 (Statistical Package for the Social Sciences, Chicago, IL, USA) was used for statistical analyses.

## 3. Results

Focusing on the main baseline characteristics of BC survivors (i.e., clinical data, educational level, employment status, current or previous practice of physical/sporting activities), the majority of the women had undergone breast-conserving surgery (47.4%), followed by a mastectomy with reconstruction (42.1%) and modified radical mastectomy (10.5%). The adjuvant treatment strategy included endocrine therapy (73.7%) and/or chemotherapy (52.6%). More than half of the women (58%) had attained a university education, 32% had a high school degree and 10% had a middle school degree. Most of the women (36.9%) were employees or housewives (21%), whereas 15.8% were freelancers, 10.5% health professionals, 10.5% social workers and 5.3% retirees. Concerning regular physical/sporting activities, 89.5% of survivors participated in physical activities that are proposed by the Ce.Ri.On. center to BC survivors in disease-stabilized outcomes, as previously described [2] (i.e., 47.5% yoga, 36.8% adapted physical activity, 21% Nordic walking, and 15.8% dragon boat). Moreover, 84.2% of women had also practiced regular physical activity before a diagnosis of BC. As far as the reasons for participating in this sailing experience are concerned, 57.9% of women took part to have a group experience, while 36.8% wanted to have a new experience.

Table 1 shows the questionnaire data at baseline and follow-up. A significant increase in the mean SF-12 mental component score was observed at follow-up, while no difference between baseline and follow-up was detected for the SF-12 physical component (Table 1). In addition, both the mean anxiety state and anxiety trait components of the STAI-Y questionnaire were significantly lower at follow-up than at baseline (Table 1). A significant decrease in the distress thermometer was also found at follow-up (Table 1).

As far as the assessment of sailing experience satisfaction is concerned, follow-up data showed that 100% of BC survivors considered sailing practice a positive experience. Although 58% of participants indicated that sailing was a strenuous physical activity, all women reported that they would repeat it again. Finally, the sailing experience corresponded to expectations for 94.7% of BC survivors.

## 4. Discussion

In this pilot study, we provide the first direct evidence that a one week tailored crew sailing experience can significantly ameliorate the QoL and PD of BC survivors. On the basis of these encouraging observations, it can be speculated that sailing practice may positively impact on the psychological well-being of BC survivors, as previously reported for the practice of dragon boating [9,10,30,31]. In particular, it is important to consider that this tailored sailing intervention had peculiar characteristics as it was a group intervention, it took place in a naturalistic outdoor place (i.e., the sea of Sardinia) far from a care center, and the proposed sporting activity was motivated and facilitated by a multidisciplinary team. In addition, sailing is an adventure sporting activity with a high emotional impact. Given all these features, we can speculate that in BC survivors the practice of sailing might help in reducing PD and improving QoL, possibly representing an alternative tool to reinforce the personal and social competencies and transform the fear of illness into a personal challenge. Therefore, though people may be reluctant to start the practice of sailing, which is commonly considered elitist and expensive [16], our preliminary observations suggest that BC survivors should be encouraged to participate in this team sport. To further strengthen our findings, it is interesting to note that while, as expected, at baseline the mean SF-12 mental component score of BC survivors was lower than the reference data from the Italian general healthy population [26], at follow-up the difference was markedly reduced. Notably, we observed a significant increase in the mean SF-12 mental component score at follow-up, while no difference between baseline and follow-up could be detected for the SF-12 physical component. This may be consistent with the fact that 58% of BC survivors indicated that the sailing experience involved strenuous physical activity. Furthermore, it is likely that a short, one week, experience might have been sufficient to achieve an improvement in the mental component, but not in the physical one.

We obviously recognize that this preliminary investigation has some intrinsic limitations, such as the relatively small BC survivor sample size and the study design exclusively based on self-report assessments. Nevertheless, it should also be considered that health psychology research studies are mainly based on data collected through questionnaires or surveys [7,32]. According to the relevant guidelines [33], we have described herein a pathway developed by an expert panel of practitioners and researchers in the field of exercise and rehabilitation in oncology to support the transition of BC survivors from health care to physical/sporting activities. On the basis of the present encouraging results, such an approach could also be applied to people with other types of cancers in future studies. Of note, we should consider that the baseline characteristics of the women (e.g., high educational qualification, physically active lifestyle, low PD) might have favored their participation in the sailing experience, thus suggesting a partial self-selection of the study group among overall BC survivors. However, this may be consistent with the current concept of personalized medicine [34]. Indeed, the present experience might represent a useful working model for future tailored psychophysical rehabilitation interventions for BC survivors based on personal exercise preferences, likely favoring a better long-term exercise adherence as reported in other contexts [7,35,36]. A further limitation of our study is that we cannot fully attribute the results to the specific sailing experience owing to the lack of a control group for comparison. Moreover, the large range of time from surgery within the study group should be considered, since we cannot exclude that it could have somehow influenced the outcomes. Of course, larger studies with longer follow-up periods will be required before definitive conclusions can be drawn, and BC survivor-specific recommendations can be made.

## 5. Conclusions

In summary, despite the aforementioned limitations, the comparison of baseline and follow-up questionnaire data revealed an improvement in the QoL and a reduction in PD of BC survivors following the one week sailing experience. Because of the overall positive impact observed, our encouraging, though preliminary, results suggest that sailing practice could represent a feasible intervention to increase the psychological well-being of BC survivors. This small pilot study may represent the necessary groundwork to design future sailing-based psychophysical interventions for BC survivors. Clearly, this kind of research is mainly aimed to inform oncological rehabilitation medical professionals, clinical psychologists, and academics about the importance of physical activity for health. Such knowledge, indeed, appears necessary to develop increasingly effective interventions with the aim of improving physical activity promotion and participation, ultimately improving the health and QoL of BC survivors.

## Figures and Tables

**Table 1:** Mean scores of the quality of life, State/Trait-Anxiety Inventory form Y (STAI-Y) and distress thermometer questionnaires of breast cancer survivors (*n* = 19) at baseline and at follow-up after a one week sailing experience.

Variables	Baseline Mean (SD)	Follow-Up Mean (SD)	*T*	Df	*p*-Value *	Effect Size (Cohen’s D)
Quality of life (SF-12)						
Physical component	51.11 (7.41)	52.07 (6.55)	−0.663	18	0.51	0.14
Mental component	44.20 (9.77)	48.37 (9.58)	−2.119	18	0.04	0.43
STAI-Y						
Anxiety state	38.71 (8.08)	34.48 (7.82)	2.701	18	0.007	0.53
Anxiety trait	39.19 (6.86)	36.81 (7.52)	1.968	18	0.03	0.33
Distress thermometer	3.37 (2.65)	2.05 (1.71)	2.150	18	0.04	0.59

Abbreviations: Df, degrees of freedom; SD, standard deviation of the mean; SF-12, Short Form-12 questionnaire; STAI-Y, State/Trait-Anxiety Inventory form Y questionnaire. * Student’s *t*-test for paired data.
